# Addressing Exposome: An Innovative Approach to Environmental Determinants in Pediatric Respiratory Health

**DOI:** 10.3389/fpubh.2022.871140

**Published:** 2022-06-14

**Authors:** Giuliana Ferrante, Salvatore Fasola, Giovanna Cilluffo, Giorgio Piacentini, Giovanni Viegi, Stefania La Grutta

**Affiliations:** ^1^Department of Surgical Sciences, Dentistry, Gynecology and Pediatrics, Pediatric Division, University of Verona, Verona, Italy; ^2^Institute of Translational Pharmacology, National Research Council, Palermo, Italy; ^3^Institute for Biomedical Research and Innovation, National Research Council, Palermo, Italy; ^4^Department of Earth and Marine Sciences, University of Palermo, Palermo, Italy; ^5^Institute of Clinical Physiology, National Research Council, Pisa, Italy

**Keywords:** children, environmental health, epidemiology, exposome, respiratory health

## Abstract

Developmental age is particularly vulnerable to impacts of environmental exposures. Until recent years, the field of environment and child health has predominantly relied on the study of single exposure–health effect relationships. The exposome is an emerging concept in epidemiology, encompassing the totality of the exposures experienced by an individual throughout life and their changes over time. This innovative approach provides a risk profile instead of individual predictors. Exposome research may contribute to better understand the complex relationships between environmental exposures and childhood respiratory health, in order to implement prevention strategies and mitigate adverse health outcomes across the life span. Indeed, an accurate assessment of the exposome needs several measurements as well as different technologies. High-throughput “omics” technologies may be promising tools to integrate a wide range of exposures. However, analyzing large and complex datasets requires the development of advanced statistical tools. This narrative review summarizes the current knowledge on exposome-based approaches in pediatric respiratory health. Further, it explores practical implementation, associated evidence gaps, research limitations and future research perspectives.

## Introduction

The pathogenesis of multifactorial chronic diseases involves complex interrelationships among genetic factors and exposures occurring throughout the life span (1). Indeed, understanding the role of the various environmental factors contributing to chronic diseases is challenging, mainly due to limitations in investigation and measurement tools. Since environmental factors generally act in synergy with other exposures and/or biological/behavioral factors, the traditional methods of analysis are unable to capture the whole burden of exposures and their impact on health (2). In addition, environmental exposures have been usually evaluated at a single time point, whereas emerging evidence suggests that their impact on human health throughout life is dynamic and that crucial time “windows of susceptibility” exist (3).

Respiratory allergic diseases like asthma and rhinitis frequently start in childhood. The specific contribution of genetics to their pathogenesis has been highlighted by many studies in the last decade (4–9). We have recently proposed a multi-factorial model characterized by a complex interplay between genes and environment (10). Indeed, a wide range of environmental risk factors have been associated with asthma (e.g., infections, allergens, urbanization, air pollution, etc.). In addition, studies on lung function deficits tracked from childhood to adulthood have identified lung function development as a key determinant for long-term respiratory health (11, 12). Thus, it is crucial to identify the environmental hazards that may affect lung function in childhood. Nonetheless, most epidemiological studies have focused on a single or a limited number of environmental factors so far, and it is still hard to catch the contribution of several environmental exposures acting in synergy (13).

**Figure 1:**
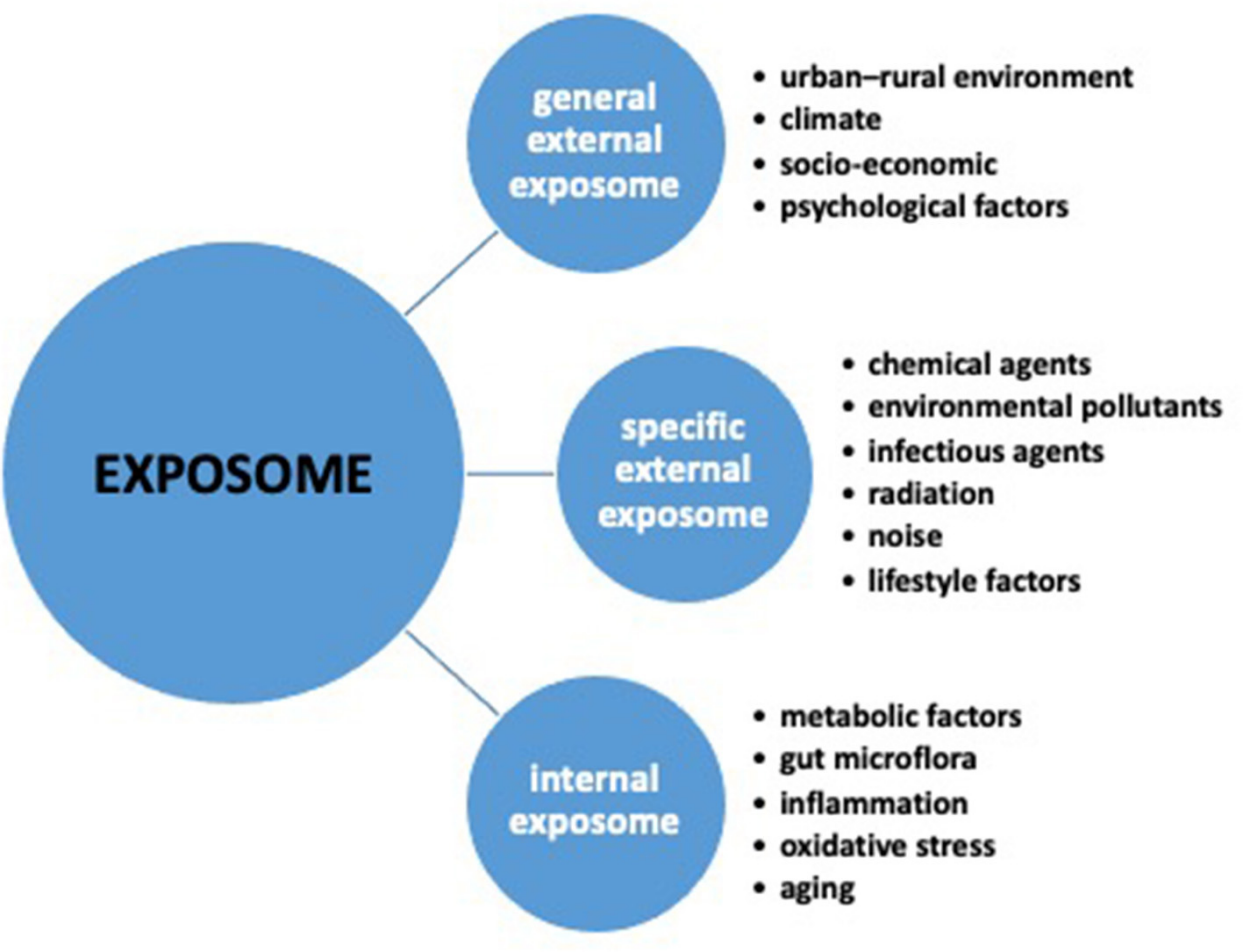
The three exposome domains: general external, specific external and internal.

In 2005, the exposome concept was introduced in epidemiology as an innovative approach for simultaneously assessing environmental risk factors and their impact on human health, thus encompassing the totality of the environmental exposures occurring throughout life (14–16). In the context of environmental health, the exposome has the ambition to cover the time frame of lifelong exposure history, and providing an accurate assessment of the impact of the environment on human health (13). As a matter of fact, the exposome concept encompasses three different domains (Figure 1): the *general external exposome*, including the urban–rural environment, climate, socio-economic, and psychological factors; the *specific external exposome*, including exposures such as chemical agents (including environmental pollutants), biological agents (infectious organisms, diet), physical agents (radiation, noise), and lifestyle factors; the *internal exposome*, including internal biological factors, such as metabolic factors, gut microbiota, inflammation, oxidative stress, and aging (14, 15, 17).

Aiming at expanding knowledge about the exposome approach in pediatric environmental health, many initiatives were subsequently launched. In 2014, the European Commission funded the “Health and Environment-wide Associations based on Large population Surveys” (HEALS) project (FP7-ENV-2013- 603946 http://www.heals-eu.eu/). The project was aimed at describe the internal exposome at the individual level by integrating omics data and biomonitoring data in order to predict the associated health outcomes in a series of about 335,000 individuals, covering different age, gender and socio-economic status groups through population studies tackling different levels of environmental exposure, age-specific windows of exposure, and genetic variability (18–20).

Also, the “European Environmental Exposure Assessment Network” (EIRENE) project (https://www.eirene-ri.eu) aims to support a comprehensive research on human health and the environment, based on the Czech national research infrastructure RECETOX (https://www.recetox.muni.cz/en/services/recetox-ri). Nowadays, EIRENE connects 50 research institutions from 17 countries, including the United Kingdom and the United States of America, with the mission of supporting research on the effects of long-term exposures to various types of stressors on population health and the roles played by these exposures in the development of chronic diseases.

In addition, within the context of the “European Long-term Study of Pregnancy and Childhood” (ELSPAC) (https://www.elspac.cz/index-en.php), a prospective study launched by the World Health Organization (WHO) in the early 1980s in six European countries and coordinated by Bristol University, UK, the CELSPAC platform (https://www.recetox.muni.cz/en/services/celspac-population-studies/celspac-study) continues to expand data collection, build large databases, and create new protocols for addressing the exposome concept in environmental health (21).

This narrative review aims to summarize the current knowledge provided by exposome-based approaches in respiratory outcomes (wheezing/asthma/rhinitis) and lung function in childhood, exploring the most relevant issues for its practical implementation, the associated evidence gaps, the research limitations, and the future research perspectives.

## Approaching Pediatric Respiratory Outcomes in Environmental Health

Among several factors such as mode of delivery, breastfeeding, mother's diet, antibiotics and other drug usage during pregnancy and early childhood, early-life environmental exposures like toxicants may significantly influence the epigenetic regulation of the immune system, which has been associated with allergic respiratory diseases (22).

With regard to respiratory allergic outcomes and lung function in children aged 2–12 years, few studies have encompassed a wide range of environmental exposures, and only two recent papers have assessed the effect of time-varying exposures on these outcomes. Rivera Rivera et al. have investigated the joint effects of prenatal and early life exposure to particulate matter <2.5 μm in diameter (PM_2.5_) and prenatal environmental tobacco smoke (ETS) on respiratory morbidity in 536 children enrolled in the “Programming Research in Obesity, Growth, Environment, and Social Stressors” (PROGRESS) study in Mexico City. The authors have identified a sensitive window between prenatal week 14 and postnatal week 18, during which PM_2.5_ exposure had been associated with a higher risk of ever wheeze at age 6–8 years, and a critical window of PM_2.5_ exposure between postnatal weeks 6 and 39 associated with a higher risk of current wheeze. Interestingly, significant associations between prenatal/early PM_2.5_ exposures and ever/current wheeze have been found only in children whose mothers had been exposed to ETS during pregnancy (23). The study highlights how taking into account concomitant environmental exposures may help characterize thoroughly some determinants of respiratory health, supporting behavioral or policy measures for reducing exposure.

Bose et al. (24) have attempted at identifying sensitive prenatal windows of exposure to nitrate (NO${}_{3}^{-}$) on lung function at the age of 7 years, considering effect modification by fetal sex, in 191 mother-child dyads. The study has found an early sensitive window during prenatal 6–12 weeks, when increasing NO${}_{3}^{-}$ was significantly associated with reduced forced expiratory volume in 1 s (FEV_1_) z-scores, especially among boys. Similar sex-specific patterns have been observed for forced vital capacity (FVC) (24). By identifying sensitive time windows within pregnancy that are more susceptible to specific environmental risk factors, these findings suggest that nitrate exposure during this early period of development may alter lung function by early childhood. Therefore, this study provides evidence linking prenatal air pollution exposures to childhood respiratory outcomes.

The first study approaching respiratory health in early childhood through the lens of the exposome has been based on the Kingston Allergy Birth Cohort (KABC), a prenatally recruited cohort with large variety in environmental exposures. Data about respiratory symptoms of 235 children at 2 years of age were obtained by parents. All the three exposome domains had effects on the respiratory health of the study population. In particular, significant associations were observed between wheeze or cough without a cold and prenatal cigarette smoke exposure, mold or dampness in the house, and the use of air fresheners in the early-life home environment. Conversely, breastfeeding, older siblings, and increased gestational age were associated with decreased respiratory symptoms (25).

The “Human Early-Life Exposome” (HELIX) project (www.projecthelix.eu), involving six prospective birth cohorts, aimed to investigate, with omics markers, the relationships between early life exposome and health in up to 32,000 mother-child pairs, as well as to measure growth, development, and health of the children, including asthma and lung function (26, 27). Within this project, the study by Agier et al. (28) investigated the association between 85 prenatal and 125 postnatal environmental exposures and lung function in children. Among 1,033 children aged 6–12 years, lower values of FEV_1_ were associated with prenatal perfluorononanoate and perfluorooctanoate, whereas the inverse distance to the nearest road during pregnancy was associated with a higher FEV_1_. Lower FEV_1_ was also associated with 9 postnatal exposures (copper, ethyl-paraben, phthalate metabolites concentrations, house crowding, and facility density around schools) (28). Again, within the HELIX project, Granum et al. (29) analyzed the associations between 90 prenatal and 107 childhood exposures and allergy-related outcomes in 1,270 children aged 6–11 years. In prenatal multi-exposure models, a lower distance of residence from the nearest road and a higher di-iso-nonyl phthalate level were associated with an increased risk of rhinitis, whereas increased particulate matter (PM_2.5_) absorbance (PMabs) was associated with a decreased risk (29). This latter finding may seem conflicting; however, PMabs, which is a measure of soot particles, can be associated with vehicle traffic as well as with other primary combustion sources not related to traffic (e.g., wood-burning, coal-burning, heating, industry). This could help explain the heterogeneity of the results. Overall, the study adds further evidence about the role of early environmental exposures on children's respiratory health and highlight the need for developing measures aimed to decrease exposure levels to prevent adverse outcomes, which in turn may have benefits in the long term.

## Challenges Related to Measurement and Data Analysis to Support Exposome-Based Approach for Pediatric Respiratory Health

**Figure 2:**
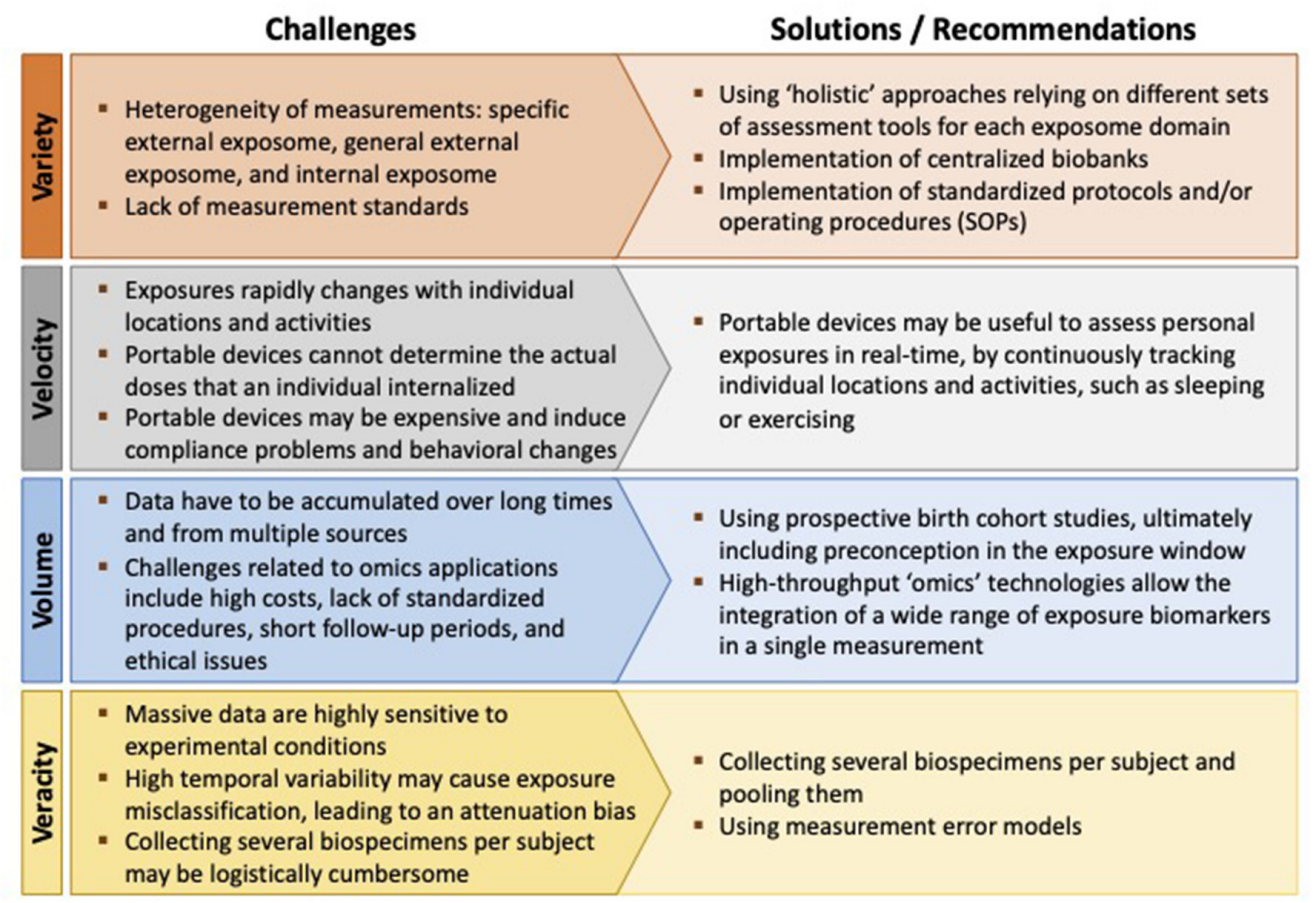
The four challenging “Vs” associated with measurement platforms required for exposomic research.

Although very appealing, addressing the exposome is challenging in terms of both measuring it and analyzing its relationship to health (13). Agache et al. (3) identified four challenging “Vs” associated with measurement platforms required for exposomic research: Variety, Velocity, Volume, and Veracity (Figure 2) (3).

The exposure assessment should be “holistic” by encompassing several aspects (Variety), and different assessment tools are required for the different exposome domains (15): conventional survey instruments or advanced sensors for personal monitoring of the *specific external exposome*, geographic information systems (GIS) for the *general external exposome*, and biomonitoring and biomarker data for the *internal exposome* (2, 13, 30). Indeed, how to integrate data across multiple methodological approaches remains a major concern (31).

Measuring the “totality” of the exposures requires the use of wearable devices able to assess personal exposures in real-time (Velocity), by continuously tracking individual locations and activities (27). It is to point out that specific activities such as sleeping or exercising may affect inhalation rates, modulating the exposure levels (3). However, challenges associated with portable devices may include high costs, compliance problems, and behavioral changes due to wearing of the monitors (3, 30). The potential issue of radiofrequency radiation is also receiving increasing attention, given the concern about exposure-related health consequences (32).

The exposome approach in allergies and lung diseases involves the collection of cumulative measures of external and internal exposures from conception (Volume), and ultimately, from preconception (2). Levels of environmental contaminants vary during lifetime, and individual changes in lifestyle can increase or decrease exposures (30). Moreover, certain life stages (for example, periods of organ development) have been identified as being more susceptible to environmental exposures with regard to asthma and allergic outcomes (3, 16). In this regard, the use of prospective birth cohort studies has been advocated (16, 33).

High-throughput “omics” technologies (lipidomics, proteomics, transcriptomics, metabolomics, etc.) allow the integration of a wide range of exposure biomarkers (both exogenous and endogenous) in a single measurement (34). However, these techniques require the ability of epidemiologists to understand the principles of omics and to identify the most useful biomarkers according to the study hypotheses (2, 30). Other challenges related to omics applications include high costs, the lack of standardized procedures, the short follow-up periods (which might not capture the dynamic nature of a disease), and ethical issues (2, 13, 35).

At last, managing to increase the volume of exposures without affecting measurement accuracy (Veracity) is another main challenge in exposome research. Indeed, massive data obtained from high-throughput platforms are highly sensitive to experimental conditions and can be easily affected by measurement error (30). Moreover, the high temporal variability of some contaminants (especially non-persistent chemicals) may contribute to exposure misclassification, possibly leading to an attenuation bias in the dose-response function (the attenuation factor is given by one minus the intra-class correlation coefficient) (13). Bias can be limited by collecting several biospecimens per subject and pooling them, but this approach may become logistically cumbersome (36).

From a statistical point of view, the most frequently used approach combines exposome-wide association study (ExWAS) and a deletion-substitution-addition (DSA) algorithm. In particular, the ExWAS incorporate estimates of individual logistic regression models independently for each exposure, in order to avoid the issue of collinearity amongst exposures. The DSA algorithm is an iterative linear regression model search-algorithm that allows selecting a reduced number of statistically significant exposures to be included in a final multi-exposure model using binomial general linear model.

Concerning the pediatric age, only three studies investigated the association among a broad range of environmental exposures and respiratory outcomes. In the study by North et al. (25), the statistical approach consisted in calculating the variance inflation factor (VIF) between each pair of prenatal or postnatal variables, and among the outcomes. Variables showing multicollinearity (VIF ≥ 10) were not included in the model, whereas variables with no evidence of multicollinearity (VIF < 10) were included in the combined model. Then, a stepwise analysis was used to explore the association between each exposure and respiratory outcomes using univariate Cox proportional hazards regression models. The study showed that all three exposome domains (Figure 1) (*general external, specific external, and internal*) influenced the respiratory health of KABC children (25).

In the study of 1,033 mother–child pairs from the HELIX cohort, Agier et al. (28) proposed the following statistical approach: the prenatal (*n* = 85) and the postnatal (*n* = 125) exposure variables were analyzed separately, using the DSA algorithm and the ExWAS. The final model was selected by minimizing the value of the root-mean-squared error of predictions by using 5-fold cross-validated data. The DSA was fitted 100 times on the data, and exposures were retained if they were selected in at least 5% of the runs. The ExWAS consisted of a covariate-by-covariate estimation of the exposure–outcome association by independent linear regression models. To correct for multiple hypothesis testing, each *p*-value was compared with a threshold, defined as 0.05 divided by the effective number of tests. All the exposure variables associated with FEV_1_% with a *p*-value of <0.20 were included in a multivariable regression model in order to account for potential co-exposure confounding (28).

More recently, Granum et al. (29) have used the HELIX cohort to evaluate the association between a broad range of prenatal and childhood environmental exposures and allergy-related outcomes in children. The statistical methods applied were: the ExWAS analyses using logistic regression model, fitted independently adjusting each *p*-value with a threshold defined as 0.05 divided by the effective number of tests, and the DSA, to select a reduced number of statistically significant exposures to be included in a final multi-exposure model (29). The authors have shown that prenatal exposure to traffic-related variables, particulate matter absorbance and phthalates were associated with rhinitis.

It is to point out that the statistical approaches used up-to-now suffer from some drawbacks: in particular, the ExWAS method cannot account for confounding by co-exposures, which is a realistic situation since people are continuously exposed to mixed exposures in their daily life. Deletion-substitution-addition overcomes the problem of adjusting for co-exposures, but it is time-consuming and highly instable due to the cross-validation procedure.

## Future Research Perspectives

The exposome has been proposed as the focus of an innovative approach in pediatric respiratory health, aiming to disentangle the contributing roles of multiple environmental factors, as well as the timing between exposures and biological responses. By providing risk profiles instead of single predictors, exposome research seems promising for a better understanding of the complex relationships among environmental exposures and childhood health, for the implementation of prevention strategies, and for mitigating adverse health outcomes across the life span.

Moreover, the exposome approach might be an innovative tool for providing new ways for addressing the underlying causes of health disparities and the biological pathways through which environmental exposures affect human health (37). Indeed, a comprehensive approach encompassing the range of social and ecological factors including housing quality, socio-economic status, and psychological stress, is crucial to examine the social-ecological context of health disparities (38). This new approach would also provide a conceptual framework useful to identify relationships between environmental exposures at critical age windows, health outcomes, and health disparities through space and time, enabling the identification of at-risk people and the targeting of public health interventions (39).

However, many gaps in this novel research field need to be carefully taken into account. First, we should be able to link exposome and genome data in order to study gene-environment interactions. Moreover, we still lack validated criteria for selecting the best assay for assessing the biological response for the chosen research question, as well as we do not yet have standards and guidelines for sample collection, repositories and biobanks (3). Data sharing and security are other issues of concern.

From a statistical point of view, there is the need for developing methodologies combining omics and exposure data, and for specific expertise to optimize the analyses of complex data involving multiple exposures, mediators, confounders, and outcomes (2).

Practical issues related to high costs associated with technological equipment in large-scale studies should also be considered. Exposome-based projects are highly expensive due to the large sample sizes required to assess multiple exposures, and to the use of innovative tools required to assess several exposome components, including environmental monitoring and omics technologies (40). Furthermore, adequate training for researchers is needed to facilitate transdisciplinary collaborations.

In summary, the following main research needs have to be addressed in the next future:

- to improve methods for identifying: the target population according to the chosen research question; the optimal study design for investigating possibly relevant associations for a particular disease outcome; the optimal timing and duration of measurements;
- to develop innovative methods for the exposome assessment;
- to establish guidelines and standards for data collection and security, as well as for the use of emerging technologies;
- to set up sophisticated bioinformatic and statistical approaches for linking a large number of exposures with biological responses and disease outcomes.

## Conclusion

In view of the recently emerging exposome concept, several European projects have started evaluating the effect of multiple environmental exposures on human health. It is expected that these projects may contribute to a better understanding of the relationships between environment and health, through a holistic approach based on the simultaneous assessment of multiple exposures.

Indeed, exposome research may help design effective interventions targeting environmental risk factors that affect respiratory health in children, and improve prevention strategies from early childhood. Nonetheless, while promising in achieving a broader view of lifetime exposures, the exposome approach is still a big challenge. The ability to measure each agent to which a person may be exposed throughout life is still limited. Furthermore, although longitudinal (prospective) cohort studies are recommended, biomarkers of exposure might not be continuously recorded for a long time.

The complexity of the factors associated with assessing the totality of the exposures, and the need to develop appropriate and effective risk management strategies should be taken into account (30). Indeed, increasing the number of time-varying exposures assessed should not result in an increased exposure misclassification, and identifying synergistic effects among exposures is crucial when interpreting study results (13). Therefore, collaboration, sustainability, and large-scale population-based studies are needed in order to improve exposome research. Such studies also need to be continuously ongoing and systematically evaluated. In addition, huge and expensive efforts are required in terms of consortia building and of development and validation of measurement devices and statistical tools.

## Author Contributions

GV, GF, and SL: conceptualization. GF, GC, and SF: writing original draft. GV, GP, and SL: review and editing. All authors read and approved the final version of the manuscript.

## Conflict of Interest

The authors declare that the research was conducted in the absence of any commercial or financial relationships that could be construed as a potential conflict of interest.

## Publisher's Note

All claims expressed in this article are solely those of the authors and do not necessarily represent those of their affiliated organizations, or those of the publisher, the editors and the reviewers. Any product that may be evaluated in this article, or claim that may be made by its manufacturer, is not guaranteed or endorsed by the publisher.
